# Experimental Pharmacology

**Published:** 1885-07

**Authors:** 


Experimental Pharmacology. A Hand-Book of Methods for Studying the Physiological Actions of Drugs. *By* L. Hermann, *Professor of Physiology in the University of Zürich. Translated, with the author's permission, with Notes and Additions, by* Robert Meade Smith, M. D., *Demonstrator of Physiology in the University of Pennsylvania. With 32 Illustrations on Wood. 12 mo., cloth, pp. 201. Philadelphia:* H. C. Lea's Son & Co. *1883.*

A most valuable little book, especially adapted for students and experimenters, which will lead many on the way to great discoveries in the field of new drugs. Only by such means as are herein employed shall we come to purify our materia medica of all trash, and test successfully the host of homeopathic drugs brought into repute after they were thrown out of the Pharmacopœia.

"The translator has attempted to elucidate the text with a careful selection of illustrations; and he trusts that his voluminous additions, which constitute nearly one-half of the entire volume, will render the work a more perfect guide to the student." (Preface),

These additions are even more practical and valuable than the original, and the American translator deserves much credit.

The make-up of the volume compares well with the other books published by Henry C. Lea's Son & Co. H. D. V.

